# *De Novo* Sequencing, Assembly, and Analysis of the Root Transcriptome of *Persea americana* (Mill.) in Response to *Phytophthora cinnamomi* and Flooding

**DOI:** 10.1371/journal.pone.0086399

**Published:** 2014-02-10

**Authors:** Bianca J. Reeksting, Nanette Coetzer, Waheed Mahomed, Juanita Engelbrecht, Noëlani van den Berg

**Affiliations:** 1 Department of Genetics, University of Pretoria, Pretoria, South Africa; 2 Bioinformatics and Computational Biology Unit, Department of Biochemistry, University of Pretoria, Pretoria, South Africa; 3 Forestry and Agricultural Biotechnology Institute, University of Pretoria, Pretoria, South Africa; Macquarie University, Australia

## Abstract

Avocado is a diploid angiosperm containing 24 chromosomes with a genome estimated to be around 920 Mb. It is an important fruit crop worldwide but is susceptible to a root rot caused by the ubiquitous oomycete *Phytophthora cinnamomi*. Phytophthora root rot (PRR) causes damage to the feeder roots of trees, causing necrosis. This leads to branch-dieback and eventual tree death, resulting in severe losses in production. Control strategies are limited and at present an integrated approach involving the use of phosphite, tolerant rootstocks, and proper nursery management has shown the best results. Disease progression of PRR is accelerated under high soil moisture or flooding conditions. In addition, avocado is highly susceptible to flooding, with even short periods of flooding causing significant losses. Despite the commercial importance of avocado, limited genomic resources are available. Next generation sequencing has provided the means to generate sequence data at a relatively low cost, making this an attractive option for non-model organisms such as avocado. The aims of this study were to generate sequence data for the avocado root transcriptome and identify stress-related genes. Tissue was isolated from avocado infected with *P. cinnamomi*, avocado exposed to flooding and avocado exposed to a combination of these two stresses. Three separate sequencing runs were performed on the Roche 454 platform and produced approximately 124 Mb of data. This was assembled into 7685 contigs, with 106 448 sequences remaining as singletons. Genes involved in defence pathways such as the salicylic acid and jasmonic acid pathways as well as genes associated with the response to low oxygen caused by flooding, were identified. This is the most comprehensive study of transcripts derived from root tissue of avocado to date and will provide a useful resource for future studies.

## Introduction

Avocado (*Persea americana* Mill.) is a diploid basal angiosperm within the Lauraceae family and contains 24 chromosomes. It is native to Central America and is a highly nutritious and oil-rich fruit grown on a commercial scale in numerous countries worldwide, including Australia, United States of America, South Africa, Chile, Spain, Columbia and Mexico. Significant losses in avocado production result from root rot caused by the soilborne oomycete *Phytophthora cinnamomi* Rands, first isolated from cinnamon trees in 1922 [1]. Phytophthora root rot (PRR) results in damage to the fine feeder roots, causing them to become necrotic and brittle. This negatively impacts water and nutrient absorption, leading to wilting, defoliation, reduced yields, and eventual tree death [2]. This hemibiotrophic pathogen is found in the majority of avocado growing areas worldwide [3] and can persist in soil or infected plant material as chlamydospores and oospores [4]. Favourable conditions cause the oomycete to sporulate and produce motile zoospores that attach to an infection site, where they invade the host tissue. *P. cinnamomi* has a wide host range and is likely to infect more than 3000 species, including avocado, pineapple, oak chestnut, eucalyptus, rhododendron and macadamia [1], [3]. This wide host range ensures that *P. cinnamomi* will persist in the environment and remain a threat to production.

Due to the damage caused by PRR, chemical control has become necessary. Phenylamides, such as metalaxyl, and the phosphonates, such as fosteyl-Al, show the best results against *P. cinnamomi* [1]. Resistance to metalaxyl has been noted [5], and production of avocado is heavily reliant on the use of phosphite trunk injections and the grafting of scions onto rootstocks tolerant to PRR. However, use of phosphite is limited to inorganic plantations and minor decreases in efficacy have been observed when *P. cinnamomi* was isolated from avocado orchards that had been previously treated with fosetyl-Al and phosphite [6]. These isolates were less inhibited by phosphite *in vitro* than control isolates [7]. The development of rootstocks tolerant to PRR is thus of great importance to commercial nurseries and is being actively researched.

PRR is often associated with moist soil conditions [8] and disease progression is accelerated under these conditions. This may be due to increased motility of zoospores and increased plant stress caused by the excess water. Wet soils promote asexual sporulation and zoospore production in *P. cinnamomi*, allowing the disease to establish and spread [1]. In addition, avocado is one of the fruit trees most susceptible to flooding or waterlogging. Low soil oxygen caused by these conditions is prevalent in many avocado productions areas but may also arise from soil-compaction or poor soil drainage. Hypoxia in flooded soils occurs as the rate of O_2_ transfer is slower through water than through air [9] and oxygen limitation is the primary cause of stress to plants in flooded soils [10]. In addition, *P. cinnamomi* growth is apparently unaffected by hypoxia [11], suggesting that the stress caused to the host due to hypoxia might be responsible for greater disease susceptibility.

Despite the adverse economic impact of PRR and hypoxia, there is still an inadequate understanding of the molecular mechanisms of the interaction between avocado and *P. cinnamomi* and the response to hypoxia. Plants exposed to multiple stresses often activate unique responses, especially when the stress is caused by both biotic and abiotic factors that elicit antagonistic responses [12], [13]. Although some avocado rootstocks show tolerance to PRR, little is known about the mechanisms resulting in this tolerance. Initial studies of the transcriptome [14] and proteome [15] have begun to explore the response of avocado to *P. cinnamomi*. However, there is a great need to investigate this relationship further and elucidate the response of the host to the pathogen in order to discern more effective control measures. The molecular response to hypoxia is also of importance if rootstocks tolerant to both PRR and low soil oxygen are to be developed. Zentmeyer (1984) suggested that the best chance to control PRR is through the development of a rootstock resistant to *P. cinnamomi* and wet soil conditions. However, studies assessing the molecular response of avocado roots to hypoxia are limited, with the majority of the studies [16], [17], [18], [19], [20] focusing instead on the response of the fruit to low oxygen conditions.

Genome and transcriptome studies are useful in elucidating complex pathways involved in plant response to stress [21], [22], [23], [24]. Despite the commercial importance of avocado, genomic resources are limited and currently the genome is not publicly available. Expressed sequence tags (ESTs) can aid in the annotation of the genome, be used in gene expression profiling, provide probes for microarray experiments, be used as molecular markers, and have several other applications. At present there are 16 558 ESTs available for avocado on the NCBI database, with the majority (>90%) originating from flower bud libraries and only a small subset derived from root tissue. ESTs represent a valuable resource to understand the molecular mechanisms involved in stress responses as they represent the transcribed regions of the genome and may thus play a more central functional role. Next-generation sequencing platforms such as Illumina, SOLiD, and Roche's 454 platforms have provided the means for gene discovery in non-model organisms. They allow the expansion of sequence databases for both model and non-model organisms at a relatively low cost.

In this study we assessed the transcriptomic response of avocado to infection with *P. cinnamomi* and flooding. RNA was extracted from root tissue of a tolerant rootstock at several time-points during three individual infection trials. Approximately 124 Mb of sequence data were generated from three separate sequencing runs utilizing the 454 platform from Roche. This data was assembled into 7 685 contigs while 106 448 sequences remained as singletons. Contigs had an average length of 614 bp. The sequence data generated represents an invaluable resource for the identification of genes involved in both disease response and those involved in response to abiotic stresses such as flooding.

## Results And Discussion

### Sequencing and assembly

**Figure 1:**
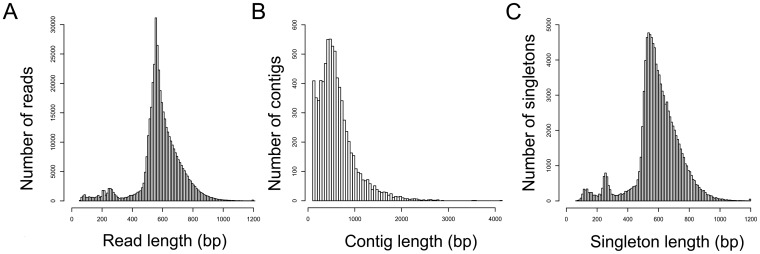
Overview of the *Persea americana* transcriptome sequencing and assembly. (A) Size distribution of raw reads. (B) Size distribution of contigs after removal of rRNA and adapter sequences. (C) Size distribution of singletons.

In order to obtain an overview of the genes involved in the response of avocado to *P. cinnamomi,* 15 cDNA libraries from a tolerant avocado rootstock either subjected to *P. cinnamomi* infection or not were sequenced in three runs on the Roche GS FLX and Titanium platforms (Table S1). Ten of the 15 libraries (L1–10) were part of a trial to assess the response of avocado to both infection and flooding. Two of these libraries included a flooding component in addition to infection and two included a flooding component without infection. The remaining six consisted of control libraries and libraries including the infection component only. The other five libraries (L11–15) only included the infection component and respective controls. Read length, contig length and singleton length were analysed to determine if the datasets were normally distributed. A Lilliefors' modification of the Kolmogorov-Smirnov statistical test was performed in R to determine if normality could be assumed. None of the datasets were found to be normally distributed. This was also true once datasets had been log-transformed. In total 436 231 reads (124.07 Mb), varying in length from 50 bp to 1201 bp (Fig. 1A), were generated and had a mean length of 585 bp (SD = 139). After trimming of adaptor sequences, 212 reads (0.05%) were removed and 436 019 reads were used in assembly. Contigs were constructed from 71% of these reads, with 56% of these reads assembling properly and 15% only partially assembling. This is close to the assembly efficiency reported in other studies [25], [26], [27]. *De novo* assembly was carried out using GS Assembler (Newbler v2.7; Roche) software. The reads were assembled into 7685 contigs (N50 = 733 bp) ranging from 100 bp to a maximum of 4121 bp (Fig. 1B), with a mean length of 613 bp (median  = 534 bp). Of the 7 685 contigs, 55% (4 211) were large contigs (>500 bp, average = 858, median = 738 bp) with a N50 = 866 bp. There were 106 448 reads that could not be assembled into contigs and that remained as singletons. These could represent low-abundance transcripts. The singletons had a median length of 589 bp (mean = 592 bp, SD = 147) and ranged in size from 66 bp to 1200 bp (Fig. 1C). The sequencing coverage of contigs ranged from 1 to 5764, with an average of 23-fold coverage (median = 8). Apart from a peak in the center, a second, smaller peak was apparent in the length distributions (Fig. 1A–C). This was because one of the three runs was performed on the GS FLX instrument, which has slightly shorter read lengths when compared to the GS FLX Titanium on which the other two runs were performed. A summary of the reads and nucleotides produced for each of the three sequencing runs is provided in Table 1. The read-depth at each position in each contig was used to calculate the average read depth for each contig and compared to contig length (data not shown). As expected, there was a positive relationship between the length of a given contig and the number of reads making up that contig (Pearson's correlation coefficient  = 0.2).

**Table 1:** Number of reads and nucleotides produced by three 454 sequencing runs of avocado root cDNA.

	Run 1	Run 2	Run 3	Total
No. of reads	10,129	347,103	78,999	436,231
No. of reads after initial quality filtering	10,126	346,996	78,897	436,019
Mean read length	248	611	517	585*
Number of bases (Mb)	2.1	91.8	30.2	124.1
Number of bases after trimming (Mb)	2.1	91.5	29.7	123.3
Total N reads assembled as contigs		329783 (76%)		
Total N reads remaining as singletons		106448 (24%)		

*Calculated from the total reads pooled from all libraries.

### Annotation

dCAS (Desktop cDNA Annotation System, version 1.4.3 Build 5240) was used to compare contigs against the NCBI non-redundant (nr) database by means of the BlastX algorithm. dCAS further processed sequences and removed redundant sequences, resulting in 7499 contigs. After annotation, contigs were manually processed and 843 (11.2%) contaminant sequences, such as microbial and ribosomal related sequences, were removed. These contigs were also compared to several unassembled *Phytophthora* datasets, including *Phytophthora sojae* (Oomycete transcriptomic database, http://www.eumicrobedb.org/transcripts/), *Phytophthora infestans* (Broad institute, http://www.broadinstitute.org), and *P. cinnamomi* (NCBI, http://www.ncbi.nlm.nih.gov/; Reitmann, unpublished). From these data a further 476 contigs were identified with homology (E-value <10^−15^) to *Phytophthora* spp, however this was brought down to 288 contigs on further analysis, leaving 6656 contigs. This subset of 6656 contigs was used in further analysis. Of these, 5830 (88%) sequences showed homology to plant sequences with an E-value <10^−5^, with 5553 (83%) having E-values <10^−10^. Of the 6656 contigs, 6140 showed homology to plant sequences in NR or Swiss-Prot while 516 had no homologous sequences. Sequences such as the latter have been reported in all plant transcriptomes and may be attributed to insufficient sequence data for the plant or closely related species. For example, transcriptomic analysis of switchgrass identified sequences not showing homology to any available sequences [28]. In this study, primers were designed for these sequences and it was shown using RT-PCR that these genes are indeed expressed in switchgrass. Singletons were also annotated but will not be discussed in detail in this paper.

### Quality evaluation of assembled sequences

In order to evaluate the quality of the assembly, we designed primers (Table S2) and confirmed amplification of nine transcripts from avocado cDNA samples (Data not shown). Furthermore, the query coverage against the NCBI nr database was assessed (Fig. S1). This gives an indication of the proportion of a contig that is matched in NCBI. In total 25% of our contigs had matches against the nr database with more than 91% coverage. More than 50% of contigs had matches with more than 71% coverage, and 75% of contigs had a match with 53% coverage or more. Select sequences were also compared to sequences obtained from the avocado genome project and showed good homology (personal communication Enrique Ibarra-Laclette).

### Functional annotation and classification

**Figure 2:**
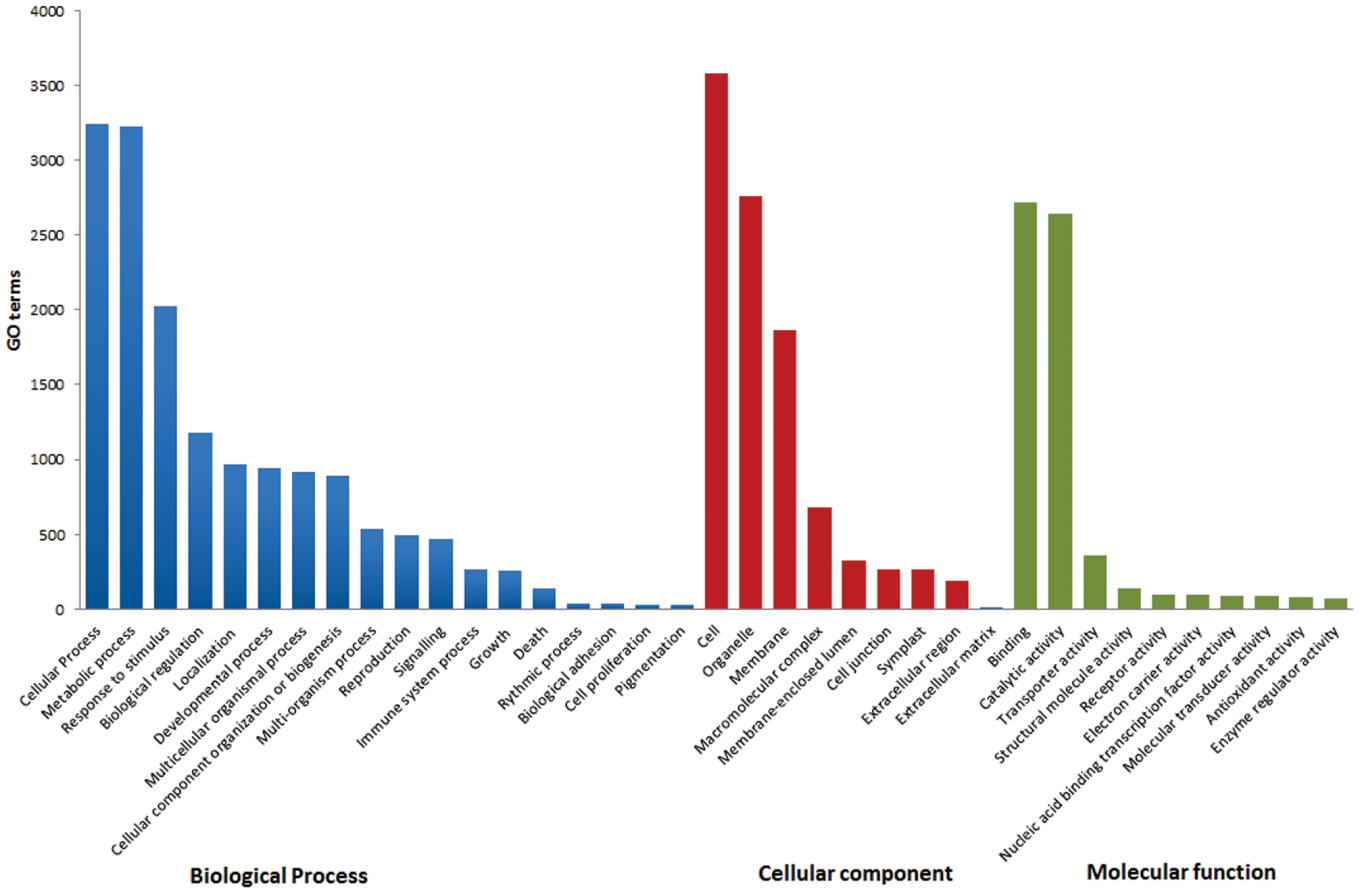
GO representation of terms within the avocado dataset. Biological process (Blue), cellular component (Red), molecular function (Green).

In order to give an assessment of the biological process, molecular function and cellular components represented by the assembly and by the individual time points and treatments, Blast2GO (B2G) analysis was conducted. B2G assigned 27981 gene ontology (GO) terms to 4924 (74%) of the contigs and used the KEGG database to assign 2221 enzyme commission (EC) numbers to 1810 (27%) of the contigs. 64 601 (61%) of the singletons had at least one GO annotation, and 11261 (11%) singletons had at least one associated enzyme code. Functional grouping of the contigs showed that within the biological process category cellular process, metabolic process, and response to stimulus were the most highly represented terms (Fig. 2). Other important terms such as biological regulation, localization, and developmental processes were also highly represented. The top three groups represented within the cellular component group were cell, organelle, and membrane related transcripts (Fig. 2). The molecular function group was dominated by transcripts with terms related to binding and catalytic activity. These results are consistent with those seen in other transcriptomic studies [29]. KEGG also assigned KEGG Orthology (KO) numbers to the contigs and assigned 2591 contigs to 273 biochemical pathways (Table 2). Of the 2591 contigs assigned to pathways, 46% of these were classified into metabolic pathways with the majority involved in carbohydrate metabolism, amino acid metabolism, and lipid metabolism. Genetic information processing was represented by 20% of the contigs and includes folding, sorting and degradation, translation, transcription, and replication and repair. Cellular processes made up 10% of the contigs. Environmental information was represented by 9% of the contigs, with signal transduction particularly well represented. Organismal systems were represented by 16% of the contigs with the sub-group environmental adaptation of interest to this study as it contains the plant-pathogen interaction category.

**Table 2:** KEGG biochemical mappings for *Persea americana*.

KEGG Pathways	Sub-pathways	Contigs	KO dentifiers
Metabolism	Carbohydrate metabolism	304	216
	Energy metabolism	192	139
	Amino acid metabolism	173	132
	Lipid metabolism	108	85
	Nucleotide metabolism	70	55
	Biosynthesis of other secondary metabolites	67	47
	Metabolism of cofactors and vitamins	56	42
	Metabolism of other amino acids	59	33
	Xenobiotics biodegradation and metabolism	85	33
	Glycan biosynthesis and metabolism	31	30
	Metabolism of terpenoids and polyketides	35	27
Genetic information processing	Folding, sorting and degradation	211	155
	Translation	202	132
	Transcription	91	75
	Replication and repair	20	11
Cellular processes	Transport and catabolism	110	85
	Cell growth and death	80	45
	Cell communication	44	21
	Cell motility	21	9
Environmental information processing	Signal transduction	218	126
	Membrane transport	5	5
	Signalling molecules and interaction	2	2
Organismal systems	Nervous system	96	51
	Immune system	72	44
	Endocrine system	69	38
	Environmental adaptation	55	30
	Excretory system	28	20
	Digestive system	25	14
	Circulatory system	20	12
	Development	19	9
	Sensory system	23	4

**Figure 3:**
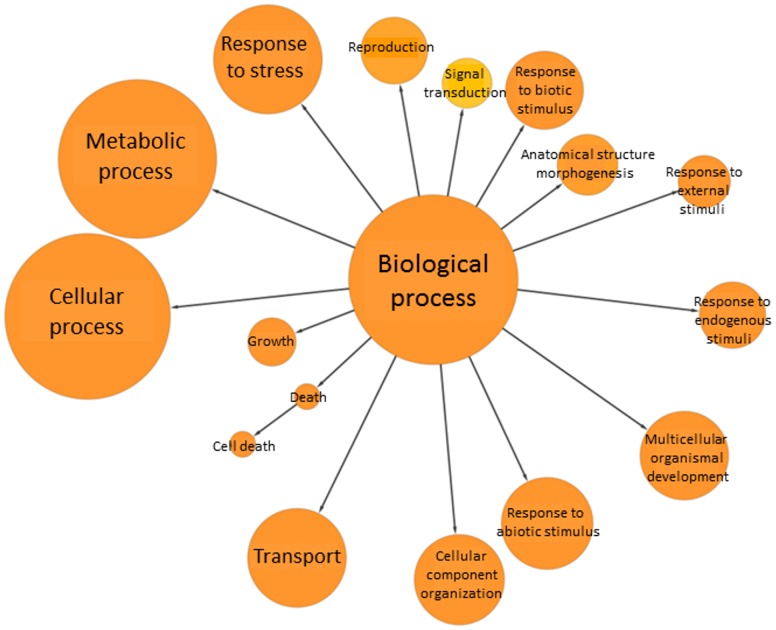
GOslim view of the avocado stress-associated transcriptome. A visual representation of enriched GO terms within the biological process category for the stress related root transcriptome of avocado. The colour and size of the nodes illustrate the significance of the GO terms. Node size represents the number of genes in each category while the node colour represents statistical significance of over-representation. (yellow – FDR  = 0.05, orange – FDR <0.05).

In order to determine what types of genes were present in the dataset, enrichment analysis was performed using GOslim for transcripts that showed more than 2-fold changes in abundance when compared to uninfected 0 hr time-points (L11 and L14) and control time-points (L1, L5, L9) (Fig. 3). Metabolic processes, cellular process, response to stress, abiotic stress stimulus, biotic stress stimulus, and endogenous stress stimulus were over represented. Changes in processes such as metabolic and cellular process are caused by the transcriptional reprogramming that takes place within the plant in response to both the pathogen [30] and abiotic stress response. Other interesting functions involved in secondary metabolic, lipid metabolic, cellular and amino metabolic processes and transport were also identified. Other GO categories that had high numbers of genes in each category were the categories for response to stress and response to abiotic stimulus. Genes within the response to stress category include genes associated with general stress response such as the heat shock proteins (HSPs), late embryogenesis abundant (LEA) proteins and universal stress proteins (USPs). Additionally, although there were fewer genes within the biotic stimulus category, these seem to be more significantly over-represented.

**Figure 4:**
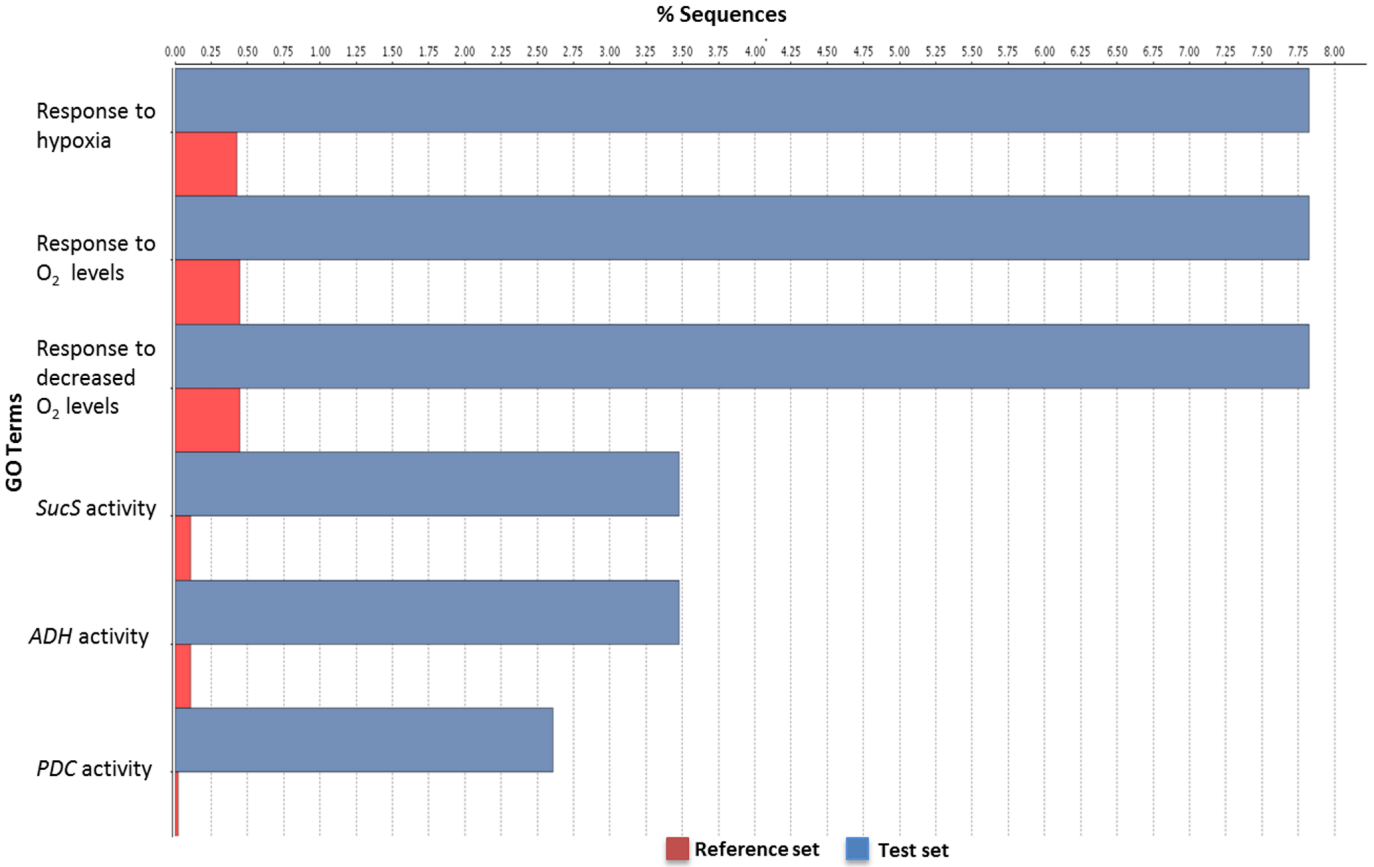
Differential GO distribution after enrichment analysis. The percentage of sequences associated with over-represented GO terms for library L3 is illustrated. Only transcripts having at least 2-fold increases in abundance were included in the analysis. (FDR <0.05).

Enrichment analysis was also performed on individual time-points and treatments to assess which types of transcripts were present at those times. Figure 4 illustrates genes showing putative up-regulation for library L3. This is 8 hrs after flooding and 7 days post-infection (dpi). The majority of transcripts enriched relate to the hypoxic response and transcripts with homology to enzymes associated with anaerobic response. *Sucrose synthase* and *pyruvate carboxylase* were found to be enriched in flooded libraries (Fig. 4) and this was validated by expression analysis conducted for these two genes (see later). All flooded libraries (L3, L6, L7, and L10) were similarly enriched for hypoxia-responsive transcripts. In later time-points water channel activity and water transport processes were putatively down-regulated (Results not shown). Although enrichment analysis can be used to determine variations in expression the purpose of the analysis in this study was to determine the categories of genes represented within each library.

### Comparative analysis with *Vitis vinifera* and *Arabidopsis thaliana*

**Figure 5:**
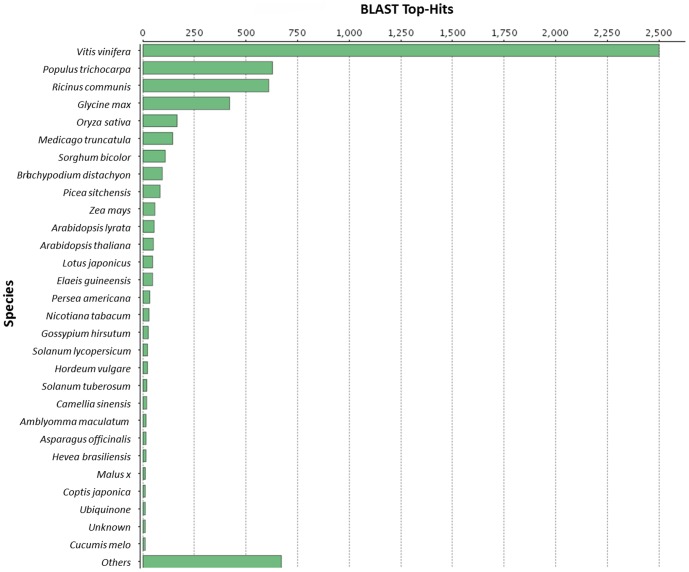
Species distribution of sequences. The majority of the avocado sequences showed homology to *Vitis vinifera*.

The highest number (41%, 2718 contigs) of avocado sequences showed homology to *Vitis spp*., with 2710 of these belonging to *Vitis vinifera* (Fig 5). Further comparisons searched for homologs to our sequences from both *Arabidopsis* (AT) and *Vitis* (VV). This was done by performing a BLAST analysis comparing the contigs to all AT and VV cDNAs from phytozome and comparing all VV sequences against AT cDNAs. The results were then compared to one another. For 5490 contigs (77%) the AT best hit phytozome annotation was exactly the same as the AT best hit annotation of the best VV hit to the contig. The AT best hit annotation was not exactly the same as the VV best hit AT phytozome annotation for 1255 contigs (18%). The remaining 400 contigs (6%) had no AT phytozome annotation. Of these 274 (4%) contigs had a best VV hit with E-value ≤0.0001, but no AT phytozome annotation for that hit. Of the 400 contigs 135 (2%) had no VV best hit with E-value ≤0.0001.

### Identification of transcripts unique to root tissue

Contigs and singletons were compared to the floral EST dataset available for *P. americana* to determine which transcripts were unique to the root and which were present in both datasets. Of the 6656 contigs, just over 51% were present in both the floral and the root datasets (Fig. S2). The unassembled singletons were also compared to the floral ESTs and it was observed that 21 201 of the 106 448 singletons (21%) were shared between the two datasets. Transcripts showing no significant homology to ESTs within the floral database were then compared to several cDNA libraries from *Vitis vinifera* (Table S3). These libraries represented several different tissues in order to further assess tissue-specificity. There were 1893 ‘root-specific’ contigs showing no significant homology to any sequences within this dataset. GO terms were assigned to 1617 of these contigs. Gene-enrichment analysis of these contigs was then carried out using B2G and categories representing drug transporter activity (GO: 0090484), drug transmembrane transporter activity (GO: 0015238), hydrolase activity (GO: 0016798), heme binding (0020037), xenobiotic transporter activity (GO:0042910), xenobiotic-transporting ATPase activity (GO:0008559), DNA integration (GO:0015074), tetrapyrrole binding (GO:0046906), and monooxygenase activity (GO:0004497) were found to be over-represented (Fishers exact test, FDR<0.1). No significant homology was seen for 72% of the singletons when compared to the avocado floral EST and *Vitis* datasets.

### Identification of abundant ESTs in avocado roots

The use of EST abundance as an indicator of transcript abundance has been validated for several studies using 454 data [14], [31], [32]. Although it is not quantitative it does give an estimate of abundance for a particular transcript under certain conditions. We identified abundant transcripts in our dataset by mapping back individual reads to assembled contigs. Seventeen contigs had more than 1000 reads mapping back (Table 3). Contig 04275 showed the highest number of reads mapping back (5 764) and showed homology (E-value = 2.25×10^−36^) to a *zinc finger protein*. Three of the highly abundant ESTs (contigs 02848, 06277, 04910) showed homology to *metallothionein or metallothionein-like proteins* (Table 3). Metallothionein proteins have been implicated in scavenging of reactive oxygen species (ROS) and the expression of genes encoding these proteins has been seen in response to abiotic stress [33]. ROS are also generated in response to pathogen attack and these proteins might play a protective role against oxidative stress. The individual libraries are shown in table 3 and the numbers correspond to the abundance ranking within each library. The first three contigs (04275, 02848, and 03342) are among the 10 most abundant transcripts across all libraries, implying that they perhaps represent transcripts which are always present in avocado roots at a high level. However, when contigs were compared against sequences (see below) produced from avocado flowers and flower, leaf, stem, and fruit tissue from *Vitis vinifera* it was observed that none of these highly abundant transcripts are unique to root tissue. Root-specific contigs with more than 100 reads mapping back to them were subsequently identified.

**Table 3:** High abundance avocado ESTs. Contigs with more than 1000 reads mapping back to them. Numbers correspond to the abundance ranking within each library*.

Contig	Reads	Gene ID	0hrs-control	0hrs-flooded (7 dpi)	8hrs-flooded (7 dpi)	8hrs-infection (7 dpi)	8hrs-control	8hrs-flooded (uninfected)	22hrs,48hrs-flooded (7 dpi)	22hrs, 48hrs-infection (7 dpi)	22hrs, 48hrs-control	22hrs, 48hrs-flooded (uninf.)	0hrs-control (uninfected)	6 hrs, 12 hrs (early infected)	24 hrs,48 hrs, 72 hrs (late infected)	0 hrs-control (uninfected)	6 hrs, 12 hrs, 24 hrs, 48 hrs (infected)
04275	5 764	*Zinc finger protein*	10	10	10	10	10	10	10	10	10	10	10	10	10	10	10
02848	5659	*Type 2 metallothionein*	10	10	10	10	10	10	10	10	10	10	10	10	10	10	10
03342	5233	*Hypothetical protein*	10	10	10	10	10	10	10	10	10	10	10	10	10	10	10
04719	3255	*Unknown*	10	10	10	10	10	10	10	10	10	10		10	10	10	10
06277	2467	*Metallothionein-like protein*	10	10	10	10	10	10	10	10	10	10	10	10	20		10
05149	2345	*Stem-specific protein tsjt1*	20	10	10	10	10	10	10	10	10	10	30	10	10	10	10
01410	2261	*Unknown*	10	10	10	10	10	10	10	10	10	10	10	20	20	30	10
07563	1895	*Unknown*	20	20	20	30	10	20	20	10	20	20	10	10	10	20	10
00150	1549	*Isoflavone 2 -hydroxylase-like*	20	20	20	10	20	10	10	10	10	10		50		20	10
04898	1524	*afg1-like protein*	30	20	20	20	30	20	20	20	20	20		10	50	10	10
04910	1511	*Metallothionein-like protein*	10	10	10	10	10	10	20	10	10	10	10	10	10		10
07570	1274	*Unknown*	10	10	10	10	20	10	20	10	10	20	30	50	10		20
00639	1083	*Zinc finger ccch domain-containing protein 49-like*	20	20	30	20			30	20	20	40		40		10	20
00331	1078	*Fructose-bisphosphate aldolase*	50	40	40	50		30	30	30		20		10	20	20	20
07526	1071	*Unknown*	10	10	10	20	20	20	30	20	20	20			30		20
01489	1059	*Aquaporin protein*	50													30	20
04136	1053	*Stress-associated ER protein 2-like*	40	30	30	40			50	30	20	40	30	10		10	20

*The number 10 implies that the particular contig was one of the ten most abundant contigs in that library, while the number 50 implies that the contig was one of the 50 most abundant contigs in that library. Dark blocks with no numbers indicate the contig was not within the 50 most abundant contigs within that specific library.

Twenty-four root-specific contigs were initially identified, with nineteen (Table 4) showing significant homology to sequences within the NR database. Several contigs coding for *extensin-like proteins* and *hydroxyproline-rich glycoproteins* were present within this set. Both classes of proteins form part of the cell wall component and have been implicated in strengthening the cell wall in plant defence [34]. *Alpha-dioxygenases* have been seen to be expressed in response to pathogens, salicylic acid, and ROS and are involved in the production of oxylipins, which have been implicated in signalling of biotic and abiotic stresses, defence against *P. infestans* [35]. A contig showing homology to a predicted *F-box/kelch-repeat protein* (At2g44130) was also amongst the abundant root-specific contigs. Expression of this gene has previously [36] been associated with increased susceptibility to root-knot nematode in *Arabidopsis* and may be involved in the defence pathway in avocado. *Pherophorin-dz1 protein* which is involved in assembly of the extracelllar matrix was also identified and has been seen to be induced in response to wounding [37]. *Beta-1,3-glucanase/glucan endo-1,3-beta-glucosidase* was represented by two contigs and has been associated with resistance against the oomycetes *Sclerospora graminicola* and *P. infestans* [38]. Nine contigs represented uncharacterized, unknown or hypothetical proteins and the five contigs not showing homology to any publically available sequences may represent avocado-specific genes or lack of sequence data for these transcripts in other species.

**Table 4:** Putative root-specific genes showing high abundance (>100 reads) in avocado roots.

Contig	Putative identity	E-value	Species
07167	*Extensin-like protein*	5e^−24^	*Vigna unguiculata*
02843	*Tyrosine-rich hydroxyproline-rich glycoprotein*	3e^−91^	*Petroselinum crispum*
05769	*PREDICTED: uncharacterized protein*	6e−^59^	*Vitis vinifera*
06691	*Predicted protein*	4e−^17^	*Vitis vinifera*
03852	*Hydroxyproline-rich glycoprotein, partial*	1e−^11^	*Phaseolus vulgaris*
00559	*Beta-1,3-glucanase*	1e^−114^	*Vitis vinifera*
00542	*PREDICTED: glucan endo-1,3-beta-glucosidase*	1e^−133^	*Vitis vinifera*
02008	*PREDICTED: F-box/kelch-repeat protein At2g44130*	5e^−77^	*Vitis vinifera*
06770	*Extensin-like protein*	2e^−34^	*Vigna unguiculata*
05072	*Predicted protein*	2e^−19^	*Populus trichocarpa*
03075	*Unnamed protein product*	2e^−20^	*Vitis vinifera*
04188	*Proline-rich extensin-like family protein*	5e^−80^	*Arabidopsis thaliana*
03185	*Unnamed protein product*	4e^−63^	*Vitis vinifera*
01428	*PREDICTED: uncharacterized protein*	2e^−95^	*Vitis vinifera*
03419	*Pherophorin-dz1 protein*	9e^−66^	*Volvox carteri*
02981	*Unknown [Populus trichocarpa]*	5e^−21^	*Populus trichocarpa*
00027	*Hypothetical protein*	0	*Sorghum bicolor*
07588	*PREDICTED: uncharacterized protein*	8e^−09^	*Vitis vinifera*
00047	*Pathogen-inducible alpha-dioxygenase*	0	*Nicotiana attenuata*

### Putative stress-related transcription factors

Developmental and stress-induced changes are regulated by transcription factors (TFs) that interact with specific *cis*-regulatory regions within the genome. These TFs fall into large gene families such as the Ethylene Response Factor (ERF), basic Helix-Loop-Helix (bHLH), MYB, basic leucine zipper (bZIP), and Zinc Finger families [39]. Several contigs within the avocado dataset show homology to these TFs (Table S4). This includes ERF TFs that bind to regions within the promoters of several stress-responsive genes such as *Pathogenesis-Related* (*PR*) genes and genes responsive to dehydration, low temperature [40] and anaerobiosis [41]. Sixteen putative WRKY TFs were present in the dataset. These Zinc finger TFs have been found to play a role in regulation of pathogen-induced defence responses, with WRKY1, WRKY2, and WRKY3 involved in the induction of the *PR1* gene in response to fungal infection [42]. Other members of the zinc finger TF family were also present in the dataset, including a Dof-type zinc-finger protein (Table S3). These TFs regulate several stress-responsive-genes and can act in unison with bZIP TFs in the signal-induced (SA, auxin, H_2_O_2_) expression of stress-related genes [42].

The basic leucine zipper TFs are involved in several processes including abscisic acid (ABA) signal transduction, interaction with PR promoters after pathogen induction, and abiotic stress response including response to cold, drought, anaerobiosis, and wounding [43]. TGA TFs belong to the bZIP family and interact with *NPR1* (non-expressor of *PR* genes) and regulate induction of *PR* genes [44]. Other bZIPs have the ability to bind to the promoter elements of anaerobiosis proteins (ANPs) and regulate expression of these genes [45]. Several contigs within the avocado dataset showed homology to these TFs (Table S4). Other TFs identified included MYBs and NAC-domain containing proteins, both of which are involved in the response to both biotic and abiotic stress. The NAC family of TFs are specific to plants and are involved in defence responses and responses to both biotic and abiotic stress [46], including low oxygen stress [47] and defence against *Phytophthora spp*. [48].

### Phytohormone related genes

Transcripts representing the SA, jasmonic acid (JA), ABA, ethylene (ET), and auxin responses were found in the avocado data. In total 291 genes representing these pathways were identified. Forty one transcripts related to SA signalling, 64 related to JA signalling, 61 to ABA signalling, 54 to ET and 71 related to auxin were found. This is expected as previous work has indicated that the avocado defence response against *P. cinnamomi* utilizes more than one defence pathway [14] and similar results were found in other hemibiotrophic infections [49].

SA markers *phenylalanine ammonia-lyase (PAL)* and *PR2* (*β-glucanase*) were identified (Table 5) and have previously been shown to be responsive to infection in the study of wheat and *F. graminearum* [49]. SA is synthesized via the phenylpropanoid pathway and the conversion of phenylalanine to trans-cinnamic acid by *PAL* is the first step of this pathway [50]. There were five putative *β-1,3-glucanases* in the avocado dataset (Table S4) and these may play a role in callose deposition in plant cells [51] or function as PR2 proteins that break down the cell walls of pathogens [52]. Two of these showed much higher transcript abundance (>10 fold) when the infected libraries were compared to control libraries. This is consistent with studies on the *Hevea brasiliensis* and *Phytophthora meadii* interaction, where β-1,3-glucanases are induced after infection and expression remains high in tolerant plants [53].

**Table 5:** Representative putative defence-related genes present in the avocado dataset.

Contig	Gene name	EC number
**00891, 05081**	*Isoflavone reductase*	
**00778, 00993, 01827, 01890, 03192**	*Glutathione S-transferase*	EC:2.5.1.18
**00419, 05090, 05446**	*Cinnamyl alcohol dehydrogenase*	EC:1.1.1.195
**00405, 00499, 00554, 01990**	*Cinnamoyl-CoA reductase*	EC:1.2.1.44, EC:1.1.1.219
**00531, 00549, 01320, 04506**	*Cysteine synthase*	EC:2.5.1.52, EC:4.2.1.50, EC:2.5.2.51, EC:2.5.1.47
**01946**	*Elicitor-responsive protein 3*	
**02831**	*Type 2 ribosome-inactivating protein*	
**00543**	*Polygalacturonase inhibitor*	
**02526**	*Cysteine protease*	
**00619, 00855, 04084, 05744, 07016, 07665**	*Chalcone synthase*	EC:2.3.1.74
**00181, 00410, 00967, 01662**	*Phenylalanine ammonia-lyase (PAL)*	EC:4.3.1.5
**04561, 05268, 05917, 05951, 06037, 06327**	*Germin-like oxalate oxidase*	EC:1.2.3.4
**00171, 02422, 04688, 05340, 05532, 07300, 07650**	*Callose-synthase (1,3-β-glucan synthase)*	EC:2.4.1.34
**01143, 03181**	*Pleiotropic drug resistance (PDR) proteins*	EC:3.6.1.3, EC:3.6.3.41
**02818, 03172, 05970**	*CC-NBS-LRR resistance proteins*	
**00327, 00962, 04873, 04944**	*HSP90*	
**00680, 06012, 06448, 05653**	*Serine/threonine protein kinases*	EC:2.7.11.0, EC:2.7.11.23, EC:2.7.11.13, EC:2.7.11.25, EC:2.7.10.2, EC:2.7.10.0, EC:4.99.1.1, EC:1.3.1.74, EC:3.4.23.0
**01155, 01993, 02354, 02832, 03974, 04619, 05898, 06314, 06692, 06860, 06935, 07035**	*Calcium-dependent protein kinases (CDPKs)*	EC:2.7.11.0, EC:2.7.11.17, EC:2.7.10
**00535, 03015, 01261, 01014**	*Endochitinase*	EC:3.2.1.14, EC:3.2.1.17
**00542, 00557, 00559, 02106, 03461**	*β-1,3-glucanase*	EC: 3.2.1.0
**01085, 01618, 02754, 02858, 03491, 04337**	*Lipoxygenase (LOX)*	EC:1.13.11.12
**01126, 02751, 03476**	*Superoxide dismutase (SOD)*	EC:1.13.11.12
**00135, 03187, 03589, 04850**	*Catalase*	EC:1.11.1.6
**00184, 00886, 00954, 03813, 04176**	*Ascorbate Peroxidase*	EC: 1.11.1.11, EC:1.11.1.7
**01593, 01967**	*PR1*	
**06278**	*PR4*	
**01450**	*PR5*	

JA is an important regulator of response to wounding, herbivores, and necrotrophic pathogens. Enzymes from the lipoxygenase pathway are responsible for the synthesis of JA from α-linolenic acid [54] and contigs showing homology to *13-lipoxygenases* were identified (Table S4). Other enzymes involved in JA biosynthesis were also identified and include *allene oxide synthase, allene oxide cyclase, 12-oxophytodienoate reductase*, and *3-ketoacyl CoA thiolase*. *F-box proteins* important in JA signalling were also identified (Table S4). ET has a variable role in defence depending on the nature of both host and pathogen [55]. Of the 54 contigs putatively linked to the ET pathway, three represented *S-adenosylmethionine synthase* (Table S4). This enzyme is involved in ET biosynthesis and it catalyzes the conversion of methionine to S-adenosylmethionine [55]. Expression analysis of these transcripts could be used to analyse whether the ET pathway is induced or repressed by the hemibiotrophic pathogen *P. cinnamomi*.

### Defence-related genes

Transcripts coding for several proteins which were previously identified [15] to be differentially expressed in avocado in response to infection by *P. cinnamomi* were identified in this study. These include homologs to *isoflavone reductase, glutathione S-transferase, cinnamyl alcohol dehydrogenase, cinnamoyl-CoA reductase, cysteine synthase* and *quinone reductase* (Table 5). *Glutathione S-transferase (GST)* had higher transcript abundance levels in the infected libraries than in uninfected libraries, consistent with previous studies where *GST* was upregulated in the *Arabidopsis*-*P. cinnamomi* interaction [56]. Cinnamyl alcohol dehydrogenase is involved in phenylpropanoid metabolism [57]. *Chalcone synthase* was present in our dataset (Table 5). Flavonoids such as chalcones, flavones, flavonols, isoflavonoids, catechins, aurones, and anthocyanins play a role as antioxidants [58].

Many other stress-responsive transcripts were identified (Table 5 and Table S4). NPR1 is an ankyrin repeat-containing protein encoded by the *NPR1* gene and is involved in SA signalling resulting in activation of defence genes [59]. NPR1 interacts with TGA TFs in order to activate this gene expression [60]. Sequences of the TGA TF identified in this study were aligned with genome sequences from the avocado genome (personal communication Enrique Ibarra-Laclette) and were found to share high homology (E-value <10^−5^). Transcripts representing a predicted *elicitor-responsive protein 3* and a *type 2 ribosome-inactivating protein cinnamomin II* were present in avocado and may be involved with elicitins secreted by *P. cinnamomi* (Table 5). A polygalacturonase inhibitor was identified (Table 5) and may be involved in inhibiting polygalacturonases secreted by invading pathogens in order to gain access to the host cell [61].

Other putative defence-related genes identified include *cysteine protease, CC-NBS-LRR (coiled-coil motif, nucleotide-binding site and leucine-rich repeat domain) resistance proteins, HSP90 proteins, and PR proteins* (Table 5). CC-NBS-LRR proteins are coded for by plant *R* genes and LRR domain-containing proteins have previously been identified in the avocado-*P. cinnamomi* interaction [14]. *PR* genes have also been identified in response to *Phytophthora* spp. Several defence-signalling transcripts were also identified (Table 5), including serine/threonine-protein kinases and *calcium-dependent protein kinases* (*CDPKs*). CDPKs are involved in the perception of PAMPs, effectors, and hormones and increased expression has been seen in tobacco in response to ABA, JA, pathogens, fungal elicitors and abiotic stress [62].

Transcripts showing homology to major reactive oxygen species (ROS) scavenging enzymes such as *superoxide dismutase* (*SOD*), *catalase* (*CAT*), and *ascorbate peroxidase* (*APX*) were also identified in avocado (Table 5). Catalase is responsible for the conversion of H_2_O_2_ into H_2_O and O_2_, limiting ROS produced in response to infection and has previously been seen to be expressed in a liquid medium in which *P. cinnamomi* was cultured [63]. Additionally, an increase in peroxidase (POD) activity in avocado roots infected with *P. cinnamomi* has been associated with ROS generation, potentially limiting pathogen growth [63]. PODs were also identified in the *Arabidopsis-Phytophthora parasitica* interaction [64], *Arabidopsis-P. cinnamomi* interaction [65], soybean-*P.sojae* interaction [66] and *Carica papaya-Phytophthora palmivora* interaction [67].

### Genes involved in flooding

The mechanisms by which plants detect and respond to low O_2_ are not well understood. Osmosensors such as the transmembrane hybrid-type histidine kinase in *Arabidopsis* verify the existence of sensors in plants [68]. Homologs to *histidine kinases* and *histidine kinase cytokinin receptors* were present within the avocado dataset (Table S4) and have been implicated in stress response and signal transduction in plants. *Prolyl-4-hydroxylase* (*P4H1*) was also present in the dataset (Table S4) and has been suggested as an oxygen sensor [60], with overexpression of P4H1 potentially mediating and mimicking the response to low oxygen in *Arabidopsis* [65]. Another possible low O_2_ signalling molecule, *non-symbiotic hemoglobin* (*NSH*), was also identified (Table 6). Increased expression of *hemoglobin* genes in barley in response to anaerobiosis has been observed [69], [70].

**Table 6:** Putative anaerobiosis-related transcripts present within the *Persea americana* dataset.

Contig	Gene name	EC number	Reference
00493	*Phosphoglucose isomerase*	EC:5.3.1.9	[78]
03561	*Glyceraldehyde-3-phosphate dehydrogenase*	EC:1.2.1.12	[79]
02647, 00326, 03694, 02647, 06605, 02093, 00004, 00050	*Sucrose synthase*	EC:2.4.1.13	[79]
00283, 01434, 00929	*Alcohol dehydrogenase 1*	EC:1.1.1.1	[78]
02359	*Alcohol dehydrogenase class-3-like*	EC:1.1.1.284; EC:1.1.1.1	
02315, 01290	*Lactate dehydrogenase*	EC:1.1.1.27	
00069, 00088, 07154	*Pyruvate decarboxylase*	EC:4.1.1.1	
00331, 00355, 00257	*Fructose 1,6-bisphosphate aldolase*	EC:4.1.2.13	
05437, 04111, 04610, 06874	*Phophoglucomutase*	EC:5.4.2.0; EC:5.4.2.2	
00049, 02414, 04570	*Hexokinase*	EC:2.7.1.2; EC:2.7.1.4	
01717, 06547	*Phosphofructokinase*	EC:2.7.1.11	
00125, 00154	*Glucose-6-phosphate dehydrogenase*	EC:1.1.1.49	[79]
00281	*Phosphoglycerate kinase*	EC:2.7.2.3	
03279, 04692, 01113, 04106, 04969, 00857	*Glutamate decarboxylases (GADs)*	EC:4.1.1.15	
04393	*Non-symbiotic hemoglobin 2*	-	[78]
03513	*Non-symbiotic hemoglobin 1*	-	
00641	*ACC oxidase*	EC:1.14.17.4; EC:1.14.11.0	[78]
00073, 00105	*Pyruvate kinase*	EC:2.7.1.40	[79]
00693, 03242, 04973	*Alanine aminotransferase*	EC:2.6.1.2; EC:2.6.1.0	

Transcription of a subset of approximately 20 genes known as the anaerobiosis proteins (ANPs), thought to be regulated by the anaerobic response element (ARE), is induced under low oxygen [10]. Most ANPs are genes involved in glycolysis and fermentation and include *alcohol dehydrogenase* (*ADH*), *aldolase*, *enolase*, *phosphoglucose isomerase*, *glyceraldehyde-3-phosphate dehydrogenase*, *pyruvate decarboxylase*, *lactate dehydrogenase* (*LDH*), *sucrose synthase* and *glucose-6-phosphate dehydrogenase*. Transcripts representing all of these genes were present in the avocado dataset (Table 6 and Table S1). In order to assess how well our dataset represented the pathways related to response to flooding we determined how many of the glycolysis/gluconeogenesis enzymes were present in the dataset. All 10 enzymes of glycolysis were identified, however sequences representing the two additional enzymes involved in gluconeogenesis, *glucose 6-phosphatase* and *fructose 1,6-bisphosphatase*, were not present. This could be due to the increased need for energy, and thus the increase in glycolysis, associated with flooding.

Energy metabolism is severely affected by low oxygen conditions and mitochondrial respiration is inhibited. Metabolism switches from aerobic respiration to fermentation in order to increase energy production while energy-intensive processes are down-regulated. Lactate is produced when aerobic respiration switches to fermentation, resulting in a drop in cytoplasmic pH [71]. Pyruvate decarboxylase is activated by this drop in pH and catalyses decarboxylation of pyruvate to acetaldehyde. ADH then oxidizes NADPH to NADP^+^ through the reduction of acetaldehyde to produce ethanol, resulting in a shift in metabolism from lactic acid fermentation to ethanol fermentation [71]. A second mechanism, thought to be an important response to hypoxia, involves the transfer of the α-amino group from glutamate to pyruvate by alanine aminotransferase (AlaAT) to form alanine [72]. Homologs to *AlaAT* were also identified within the dataset (Table 6). Other genes in the dataset related to hypoxia included *calcineurin B-like* (*CBL*)-*interacting binding kinases* (*CIPKs*), *phospholipase C* (*PLC*), *NADPH oxidase*, *Rop GTPase activating protein* (*RopGAP4*), *Rho of Plants* (*ROP*) *G-proteins*, *phosphoenolpyruvate carboxylase* (*PEPC*), *malate dehydrogenase* (*MDH*) and *nitrate reductase* (Table S4 and Table 6). [72],

### Quantitative expression analysis

Real-time expression analysis was conducted for several genes to determine if transcript abundance could be correlated to gene expression. Although sequence depth was not great enough to determine quantitative expression, expression of nine of 13 genes correlated to the sequencing data. These included six of nine genes quantified for libraries L11–L13, and four genes quantified for libraries L1–L10. The expression of the first nine genes is described in Mahomed and van den Berg 2011 and will not be discussed further. Expression analysis for the libraries assessing the response to both flooding and *P. cinnamomi* involved time-point analysis of *sucrose synthase, non-symbiotic hemoglobin, pyruvate decarboxylase*, and *endochitinase*. Gene expression was normalized utilizing three endogenous control genes, namely *Actin, 18S*, and *alpha-1 tubulin*.

**Figure 6:**
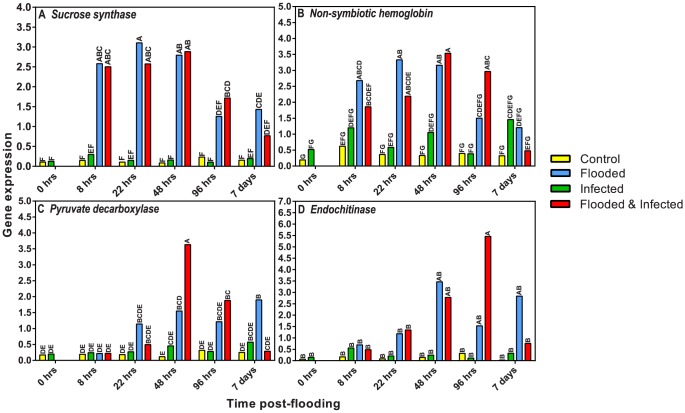
Relative expression levels of four avocado genes. *Sucrose synthase* (A), *non-symbiotic hemoglobin* (B), *pyruvate decarboxylase* (C), *endochitinase* (D). Bars represented with the same letter are not significantly different at P<0.05. The x-axis represents time after flooding was commenced.

Expression of *sucrose synthase* was significantly up-regulated in both flooded treatments by 8 hrs after commencement of flooding (Fig 6A). High levels in these treatments were maintained up until 96 hrs when increases in the uninfected, flooded treatment were no longer significantly different from the non-flooded treatments. The flooded and infected treatment remained significantly higher than the non-flooded treatments up until 7 days when differences were no longer significant. *Sucrose synthase* expression is clearly induced by flooding and appears to be unaffected by infection as control and infected non-flooded plants showed no differences in expression (Fig. 6A). Although later time-points (96 hrs, 7 days) were not included in the sequencing, the earlier time-points show a correlation between expression and transcript abundance for this transcript. Non-flooded libraries show lower abundance in the 8 hr libraries whilst no transcripts could be mapped back to contigs originating from the 22 hrs/48 hrs non-flooded libraries. All flooded libraries had at least 12–19 reads mapping back to the contig representing *sucrose synthase*.

Expression of *non-symbiotic hemoglobin* was also assessed. This gene was selected for its suggested role in signalling low oxygen stress as well as its low transcript abundance in the libraries in order to determine if the correlation between transcript abundance and gene expression was still valid for low abundance transcripts. The transcript abundance data indicated that the highest abundance levels were present in L6 and L10, which correlates with the expression profiles (Fig. 6B). Flooded treatments both showed increases in expression at 8 hrs, however differences were only significantly different from controls in the uninfected, flooded treatment. Differences only became significant for infected, flooded treatments at 22 hrs. These increases in expression were maintained in both treatments up until 96 hrs when the uninfected treatment no longer showed significant differences from the control. The infected, flooded treatment showed higher levels of expression up until 7 days by which levels had dropped back to control levels. The delay in expression seen in the infected, flooded treatment when compared to the uninfected, flooded treatment is interesting as it may suggest some synergistic effects between flooding and infection. Non-flooded infected plants did not show significant differences in expression from control plants, suggesting that, as observed for *sucrose synthase*, this gene is responsive to flooding and not to infection alone.

*Pyruvate decarboxylase* expression also correlated with the transcript abundance, showing the same trend of up-regulation as observed for *sucrose synthase*. The highest transcript abundance was seen in the 22 hrs/48 hrs flooded libraries (L7, L10), correlating with the expression data. However, high abundance levels were also seen in the 8 hrs uninfected, flooded library (L6) which was not observed in the expression analysis. *Pyruvate decarboxylase* activity was unaffected by infection (Fig. 6C). Increases in expression were seen in the uninfected, flooded treatment at 22 hrs and were significantly different from control levels by 48 hrs. However, differences were not significantly different from non-flooded infected plants at this point. These slightly elevated expression levels were maintained until the end of the trial. Interestingly, the infected, flooded treatment showed significant differences in expression from all other treatments at 48 hrs and maintained higher expression relative to non-flooded treatments up until 7 days when expression returned to control levels (Fig. 6C). This difference in expression between uninfected and infected flooded treatments and the similar expression patterns observed in non-flooded treatments again suggests a synergistic interaction between flooding and infection.

The final gene assessed was *endochitinase*. Transcript abundance between libraries was similar and is reflected by the qPCR results. The expression analysis for libraries that were sequenced indicated no significant differences in expression for any of the treatments or time-points assessed (Fig. 6D). Significant differences were only observed at 96 hrs for the infected, flooded treatment. However, by 7 days expression had returned to control levels. There was a trend in the data suggesting that flooding may cause slight increases in expression of *endochitinase*.

## Conclusions

Sequence data for the root transcriptome of the non-model plant avocado was generated using 454-pyrosequencing and this data has been *de novo* assembled. Despite the economic importance of this crop, the genome for avocado is not yet publicly available and sequence data for avocado is limited. We identified over 5550 contigs as putative homologs (E<10^−10^) of annotated plant sequences in public databases. Many putative defence- and stress-related homologs were present in the dataset. These genes provide a valuable resource for avocado which can be used in further studies of the avocado-*P. cinnamomi* interaction and in the response of avocado to hypoxia caused by flooding. Expression analysis of several stress-related genes was assessed and expression of three flood-responsive genes, *sucrose synthase, non-symbiotic hemoglobin*, and *pyruvate decarboxylase* was confirmed for avocado. Additionally, in accordance with literature, the expression data indicates that a synergistic relationship exists between flooding and infection with *Phytophthora cinnamomi*. Large-scale expression analysis utilizing this set of stress-related contigs will enable stress-responsive networks and pathways to be identified in avocado, both in response to biotic and abiotic factors. In addition, this set of data can be used in studies assessing other stress-responses or infection by other pathogens as the genes linked to these responses often overlap. Candidate genes for further study can thus be selected.

## Methods

### Plant material

Clonal PRR-tolerant Dusa® plantlets provided by Westfalia Technological Services (Tzaneen, South Africa) were used in all plant trials. Root material was harvested at several time-points and immediately stored at −80°C. See File S1 for details.

### *Phytophthora cinnamomi* isolates and infection

*Phytophthora cinnamomi* isolates were obtained from declining avocado orchards in Tzaneen, Limpopo, South Africa. Permission to collect isolates was obtained from individual farmers and from Westfalia Technological Services. Pathogenicity of isolates was confirmed by infecting avocado plants in a pre-trial and assessing disease development. See supplementary material for details on individual infection trials. Infection was confirmed by re-isolation of the pathogen and subsequent DNA extraction using Prepman™ Ultra (Applied Biosystems, Foster City, CA). Isolates were confirmed as *P. cinnamomi* by use of the species specific LPV3 primers (LPV3 F 5′-GTG CAG ACT GTC GAT GTG-3′, LPV3 R 5′-GAA CCA CAA CAG GCA CGT-3′) [73] in a polymerase chain reaction (PCR).

### RNA extraction

RNA was extracted from ground root tissue using the CTAB extraction method described by [74], with slight modification. The chloroform: isoamyl alcohol step was repeated 3–5 times, depending on the stability of the interphase and colour of the sample. RNA concentration and integrity was estimated using the NanoDrop® ND-1000 (Nanodrop Technologies, Inc., Montchanin, USA) spectrophotometer and non-denaturing 2% TAE agarose gels. mRNA isolation was performed using the Oligotex™ mRNA kit (Qiagen Inc., Hilden, Germany). See supplementary materials for additional information.

### cDNA library construction and 454 sequencing

Double-stranded cDNA was synthesized from purified mRNA using the Roche cDNA synthesis system (Roche, Mannheim, Germany) according to the manufacturer's instructions. First strand cDNA was generated by using either oligo dT_15_ primers or random hexamers to prime synthesis. cDNA was then purified using the MinElute PCR Purification kit (Qiagen) to remove any contaminants before sequencing. Integrity of cDNA was assessed using gel analysis. Genomic DNA (gDNA) contamination was assessed using the intron-spanning flavone-3-hydroxylase (F3H) primers, F3H F 3′-TCTGATTTCGGAGATGACTCGC-3′ and F3H R 3′-TGTAGACTTGGGCCACCTCTTT-3′ (Inqaba Biotec, Sunnyside, South Africa). Individual libraries were tagged with adapter sequences. cDNA was sequenced utilizing the Roche 454 GS-FLX and the Titanium platform (Inqaba Biotec). All sequence data generated from this study is available on the NCBI Transcriptome Shotgun Assembly Sequence Database BioProjectID: PRJNA72155.

### Assemblies

GSassembler version 2.7 (454/Roche Life Sciences) was used to assemble the sequence data into contigs using the default settings (40 bp overlap, 90% identity). The sff files were extracted using sff_extract version 0.2.13 (COMAV Institute, Universidad Politecnica de Valencia). Adapter sequences were trimmed before assembly using a custom script (Python). Read quality was assessed and low quality reads were excluded from contig assembly. BLAT version 34 [75] was used to map reads back to individual libraries. R version 2.13.2 (http://cran.r-project.org) was used to construct histograms.

### Functional annotation and characterization of ESTs

Annotation of all unique sequences (contigs and singletons) and removal of redundant contigs was carried out using dCAS. Assembled contigs were annotated using several databases including: the Gene Ontology (GO), EuKaryotic Orthologous Groups (KOG), nr, pfam, rrna, smart, univec, and mit-pla databases. Blast2GO (B2G; http://www.blast2go.com) was used to assign GO terms describing biological processes, molecular functions and cellular components. The data presented herein represent a GO analysis at level two. Enzyme commission (EC) numbers were assigned using B2G linked to the KEGG database. InterProScan was used to identify proteins based on structural characteristics and the results were then annexed with the annotations against the nr database in B2G to give an annotation of higher confidence. Enrichment analysis was performed using B2G based on the transcript abundance of each library relative to its corresponding control library. In order to get an indication of differential expression, each library was compared to the control library for that time-point after scaling each abundance value to library size [76]. Only transcripts showing differences in abundance as compared to the respective control libraries were used for enrichment analysis.

### Quality assessment

Quality of assembled contigs was assessed by designing primers and amplifying sequences from cDNA. Primers were designed using PerlPrimer v1.1.21 (http://perlprimer.sourceforge.net) and were synthesized by Integrated DNA Technologies (IDT) and supplied by Whitehead Scientific (Pty) LTD (Cape Town, South Africa). Further assessment was carried out by plotting the percentage of the length of query sequence that matches the best blast hit in NCBI. This data was obtained from the dCAS results column ‘% Match Length’. Alignments to genomic sequences obtained from the avocado genome project were aligned using MAFFT [77].

### RT-qPCR

cDNA synthesis for RT-qPCR was carried out using the ImProm-II™ single strand cDNA system according to manufacturer's instructions (Promega Corporation, Madison, USA). First strand synthesis was primed utilizing 0.5 µg random hexamers (Invitrogen Life Technologies, California, USA). cDNA quality was assessed as described above. The expression of four avocado genes was investigated using the Bio-rad® CFX 96 instrument. These genes included *sucrose synthase (SucS), non-symbiotic hemoglobin (NSH), pyruvate decarboxylase (PDC)*, and *endochitinase (Endo)*. Several genes were evaluated to determine their suitability as endogenous control genes and three genes with M values well below 0.5 were chosen. Primers for RT-qPCR were designed utilizing PerlPrimer v1.1.21 (http://perlprimer.sourceforge.net) and synthesized by IDT. Primers were designed to amplify a product of no more than 150 bp (Table S5) with melting temperatures ranging from 55–60°C. Primer specificity was tested by first performing a conventional PCR and confirmed by the presence of a single melting curve. Serial dilutions (1∶5, 1∶10, 1∶25, 1∶50, 1∶100, 1∶500, 1∶1000) were made from a pool of cDNA from all treatment groups and time-points and calibration curves were performed for each gene. Expression was normalized using three endogenous control genes; *Actin, 18S, and alpha1-tubulin*.

### Statistical analysis

A Tukey's test was performed to determine significance for quantitative gene expression analysis. Statistical analysis was carried out utilizing the JMP® program version 10.0.0. Significance was assessed at P<0.05.

## Supporting Information

Figure S1 Quality evaluation of assembled avocado sequences. Percentage of the query sequence covered by the blast hit plotted against the number of contigs.
(EPS)

Figure S2 Overlap of root transcripts with floral ESTs. Proportion of avocado contigs (A) and singletons (B) that show significant homology (E<10^−5^) to sequences in the avocado floral EST set.
(EPS)

File S1 Supplementary methods.
(DOCX)

Table S1 Summary of the libraries used in the assembly of the *Persea americana* root transcriptome.
(DOCX)

Table S2 Primer sequences used to test assembly. Nine of the sequences could be amplified from avocado cDNA.
(DOCX)

Table S3 *Vitis vinifera* cDNA libraries used to determine unique root transcripts in avocado.
(DOCX)

Table S4 Putative stress-responsive genes present within the *Persea americana* dataset.
(DOCX)

Table S5 Primer sequences for genes analysed in expression analysis.
(DOCX)
