# Supplementary material for: De Novo Sequencing, Assembly, and Analysis of the Root Transcriptome of Persea americana (Mill.) in Response to Phytophthora cinnamomi and Flooding
Source: PLoS One. 2014 Feb 10;9(2):e86399. doi: 10.1371/journal.pone.0086399 (PMC3919710; doi:10.1371/journal.pone.0086399)
Supplement: File S1 — Supplementary methods. (DOCX) [file pone.0086399.s003.docx]

**Supplementary methods**

***Plant trials***

*P. cinnamomi infection trials*

Nine-month old plantlets were inoculated with mycelia. Mycelial suspension was prepared by homogenising 33g of mycelia in 65L of distilled water to give a final concentration of 0.5g/L. This was then mixed into a mist bed containing 112kg of vermiculite. Plants were then planted in the mist bed. Previous microscopy work showed that germination of zoospores occurred within an hour after infection and hyphae were visible by 6 hours. Root material was thus harvested at 0 (uninfected), 3, 6, 12, 24, 48, and 72 hours after infection. A subset of plants was left in the mist bed for 6 weeks as a positive control for disease.

*Flooding and P. cinnamomi infection trial*

The experiment was conducted in a greenhouse with 1-year old plants. Plants were acclimatised to greenhouse conditions for 3 months before the start of the experiment. Plants were planted in 2L containers in a soil-perlite mix (1:1, v:v) and maintained in a greenhouse at the Forestry and Agricultural Biotechnology Institute, Pretoria, South Africa (25° 45' 19.63" S, 28° 14' 7.75"E). The average maximum temperature in the greenhouse was 24.9°C with an average minimum temperature of 13.7°C. Plants formed part of one of four treatments; Control, infected, flooded, and flooded and infected. Natural light in the greenhouse was supplemented between 6am and 6pm by sodium and mercury lamps, providing a 12 hour photoperiod. Plants were watered 3-4 times weekly and supplemented with Hoagland’s solution [[106](#_ENREF_106)] once a week. Plants were infected using 50ml/plant of a zoospore suspension at a concentration of 2.5×10^4^ zoospores/ml.

Zoospore suspension was made by placing blocks of colonized V8 agar (20% V8 juice (v:v), 0.25% CaCO_3_, agar 17g l^-1^) into 2% V8 broth for 3 days or until sufficient mycelial growth was evident. Mycelial plugs were then rinsed three times with dH_2_O and placed into 90 mm petri dishes filled with filtered stream water. These plates were left under UV light for 2-3 days to induce sporangia formation. Cultures were cold-shocked at 4°C for 45 min and were left at room temperature for an hour to allow release of zoospores. Infection was carried out immediately after sufficient zoospore release was evident under the microscope in order to ensure motility of the zoospores.This suspension was poured directly into the potting medium alongside the stem. Plants were flooded a week after infection with *Phytophthora cinnamomi*. Flooding was carried out using plastic reservoirs filled with tap water to 1cm below the potting mixture. Root samples were isolated from each treatment at six time-points; 0hrs, 8hrs, 22hrs, 48hrs, 96hrs, and 7 days.

***cDNA library construction and 454 sequencing***

*P. cinnamomi infection trials*

Data from a previous infection study [[14](#_ENREF_14)] were combined together with a larger sequencing event. Equal amounts of total RNA from three biological replicates (and three technical replicates) per time point was combined prior to mRNA isolation. First strand cDNA synthesis was primed using random hexamers. Briefly, the first sequencing run performed on the 454 GSFLX system consisted of three libraries that were constructed from the different time points of the infection trial and designated L11 (0hrs uninfected), L12 (6 and 12 hr pi) and L13 (24, 48 and 72 hr pi). The second run was performed on the 454 GSFLX Titanium platform and consisted of two libraries, an uninfected 0hr library (L14) and L15, an infected library (6, 12, 24, and 48 hours). Adapters were used to tag the different libraries.

*Flooding and infection trial*

RNA was treated with DNase (Thermo Scientific Inc., Bremen, Germany) to eliminate any genomic DNA contamination and purified using the RNeasy ® Mini Kit (Qiagen Inc., Hilden, Germany). Equal amounts of RNA from three biological replicates, consisting of two plants each, was combined for each library prior to mRNA isolation. Each biological replicate was obtained by pooling 20ug of RNA from two separate plants to yield 40ug of RNA. A total of 10 libraries were generated, including; (L1) 0hrs uninfected, (L2) 0hrs infected, (L5) 8hrs control, (L4) 8hrs infected, (L6) 8hrs flooded, (L3) 8hrs infected and flooded, (L9) 22hrs and 48hrs control, (L8) 22hrs and 48hrs infected, (L10) 22hrs and 48hrs flooded, and (L7) 22hrs and 48hrs infected and flooded. mRNA isolation was performed using the Oligotex™ mRNA kit (Qiagen Inc., Hilden, Germany) by loading 120 µg of RNA onto the columns. Samples were eluted in 25ul elution buffer three times using the same eluent. In addition, samples were kept at 70°C during elution. Elution conditions were optimized to ensure maximal yield. First strand cDNA synthesis was primed by oligo-dT primers. Approximately 4.5 µg of cDNA was sent for sequencing at Inqaba Biotec. Adapters were used to tag individual samples within a lane.
