# Supplementary material for: De Novo Sequencing, Assembly, and Analysis of the Root Transcriptome of Persea americana (Mill.) in Response to Phytophthora cinnamomi and Flooding
Source: PLoS One. 2014 Feb 10;9(2):e86399. doi: 10.1371/journal.pone.0086399 (PMC3919710; doi:10.1371/journal.pone.0086399)
Supplement: Table S1 — Summary of the libraries used in the assembly of the Persea americana root transcriptome. (DOCX) [file pone.0086399.s004.docx]

Table S1. Summary of the libraries used in the assembly of the *Persea americana* root transcriptome*.

| **Library** | **Stress** | **Reads/library** |
| --- | --- | --- |
| L1 | 0hrs-control | 3757 |
| **L2 | 0hrs-flooded (7dpi) | 21973 |
| L3 | 8hrs-flooded (7dpi) | 4663 |
| L4 | 8hrs-infection only (7dpi) | 12123 |
| L5 | 8hrs-control | 5726 |
| L6 | 8hrs-flooded (no infection) | 6562 |
| L7 | 22hrs, 48hrs-flooded (7dpi) | 5742 |
| L8 | 22hrs, 48hrs-infection only (7dpi) | 5749 |
| L9 | 22hrs, 48hrs-control | 6141 |
| L10 | 22hrs, 48hrs-flooded (no infection) | 6563 |
| L11 | 0hrs-control (uninfected) | 1432 |
| L12 | 6hrs, 12hrs (early infected) | 3652 |
| L13 | 24hrs, 48hrs, 72hrs-late infected | 5045 |
| L14 | 0hrs-control (uninfected) | 148874 |
| L15 | 6, 12, 24, and 48 hours (infected) | 198229 |

*Three sequencing runs were performed and the libraries from each run are separated by a dashed line.

**Library L2 is an infected, non-flooded library that serves as the 0hrs control for the flooded, infected treatments.
