# Supplementary material for: De Novo Sequencing, Assembly, and Analysis of the Root Transcriptome of Persea americana (Mill.) in Response to Phytophthora cinnamomi and Flooding
Source: PLoS One. 2014 Feb 10;9(2):e86399. doi: 10.1371/journal.pone.0086399 (PMC3919710; doi:10.1371/journal.pone.0086399)
Supplement: Table S2 — Primer sequences used to test assembly. Nine of the sequences could be amplified from avocado cDNA. (DOCX) [file pone.0086399.s005.docx]

Table S2. Primer sequences used to test assembly. Nine of the sequences could be amplified from avocado cDNA.

| **Contig** | **Forward primer (5’-3’)** | **Reverse primer (5’-3’)** |
| --- | --- | --- |
| 00181 | TTGCGAGAAGTACTAGTGGAG | ATCGAATACCCGTCTTCTTCC |
| ACT | CCAAGCAGCATGAAGATAAAGGT | CACATCTGTTGGAAGGTGCTC |
| PDC | GAGGGTGCAAACACAATGGA | CGCAATACAGTAACCCAAACCA |
| PAD4 | GACATGATCCAGAAGAACCAGC | ATATTTCCAAAGGCTCCACAAGG |
| ADH-P | AGAAACTGTCTCCTATTCGTC | CATGAACAAGGTAAATAGGCAC |
| SucS | CATACATCAAACCGTGAGATCCA | GTACTACTTGCAACCAGCGT |
| NSH | TCAAGATGACCTGTGAAGCA | CCTTCTTAAGATGAACTGAACCC |
| 04084 | ATGTGCTTAGTGAGTATGGGA | CAAACATGGATCTTTGACAACG |
| 00675 | CCAACTCGTTTGAAGCCGTC | GATAGTCGAAAGGGAGGTAGAGG |
| 02081 | CTCCAAATGAACCGTATCGT | TCTGTCTTAGGAAGGTCAGG |
| 00844 | TGTTGTGACCATCAGATCATGGA | CAAACCAGCAGAATTCCTCGG |
