# Supplementary material for: De Novo Sequencing, Assembly, and Analysis of the Root Transcriptome of Persea americana (Mill.) in Response to Phytophthora cinnamomi and Flooding
Source: PLoS One. 2014 Feb 10;9(2):e86399. doi: 10.1371/journal.pone.0086399 (PMC3919710; doi:10.1371/journal.pone.0086399)
Supplement: Table S3 — Vitis vinifera cDNA libraries used to determine unique root transcripts in avocado. (DOCX) [file pone.0086399.s006.docx]

**Table S3**. *Vitis vinifera* cDNA libraries used to determine unique root transcripts in avocado

| Biosample  ID | cDNA Library Name | Source | Tissue | No. of ESTs |
| --- | --- | --- | --- | --- |
| 150636 | Buds (VvS2) | Universidad Tecnica Federico Santa Maria | Buds | 4092 |
| 153723 | Ovule cDNA library of a seedless grape (Vitis vinifera var.Thomson seedless) | College of Horticulture, Northwest A&F University | Ovule | 4075 |
| 153981 | Cabernet Sauvignon Flower Pre-bloom - CAB1 | UC Davis | Flower | 3748 |
| 154009 | Veraison Grape Berries Lambda Zap II Library | INRA, Romieu | Berry | 263 |
| 154017 | Cabernet Sauvignon Flower bloom - CAB2 | UC Davis | Flower | 4428 |
| 154026 | CabSau Flower Stage 12 (FLOu0012) | University of Nevada | Flower | 18612 |
| 154027 | CabSau Flower Stage 17 (FLOu0017) | CSIRO | Flower | 46 |
| 154029 | CabSau Flower Stage 7 (FLOu0007) | CSIRO | Flower | 52 |
| 154266 | CabSau Immature Inflorescence Stage 31 (INFu0031) | CSIRO | Immature Inflorescence | 967 |
| 154341 | Inflorescence (VvS11) | Universidad Tecnica Federico Santa Maria | Inflorescence | 3691 |
| 154342 | Inflorescence with GA3 (VvS12) | Universidad Tecnica Federico Santa Maria | Inflorescence | 4023 |
| 738717 | Vitis vinifera cv. Pinot Noir PN177 cDNA library | Centre National de Sequencage, Genoscope | Flowers | 24419 |
| 150606 | Muscat Hamburg pre-veraison berry normalized (WIN11) | entro Nacional de Biotecnologia, CSIC | Berry | 17877 |
| 150630 | Fruits Veraison (VvS8) | Universidad Tecnica Federico Santa Maria | Fruits | 2834 |
| 150631 | Ripening Berries (VvS9) | Universidad Tecnica Federico Santa Maria | Berry | 4169 |
| 150632 | Ripening Berries with GA3 (VvS10) | Universidad Tecnica Federico Santa Maria | Berry | 79 |
| 150635 | Rippening Berries (last harvest) (VvC5) | Universidad Tecnica Federico Santa Maria | Berry | 81 |
| 153882 | Veraison Grape berries Lambda TriplEx2 Library | Universite de Bordeaux I, INRA | Berry | 398 |
| 153883 | Red Grape berries Lambda TriplEx2 Library | Universite de Bordeaux I, INRA | Berry | 337 |
| 154001 | Veraison Grape Berries Psport1 Library | Universite de Poitiers | Berry | 52 |
| 154002 | Veraison Grape Berries Lambda Triplex2 Library | Universite de Poitiers | Berry | 193 |
| 154003 | Green Grape Berries Lambda Triplex2 Library | Universite de Bordeaux I, INRA | Berry | 1214 |
| 154004 | Ripe Grape (harvested at 5 p.m.) Pedicles Lambda Triplex2 Library | Universite de Poitiers | Pedicle | 580 |
| 154005 | Ripe Grape (harvested at 9 a.m.) Pedicles Lambda Triplex2 Library | Universite de Poitiers | Pedicle | 782 |
| 154006 | Green Grape (harvested at 9 a.m.) Pedicles Lambda Triplex2 Library | Universite de Poitiers | Pedicle | 1274 |
| 154007 | Ripe Grape Berries Lambda Triplex2 Library | Universite de Poitiers | Fruit | 416 |
| 154008 | Green Grape (harvested at 5 p.m.) Pedicles Lambda Triplex2 Library | Universite de Poitiers | Fruit | 1375 |
| 154010 | Ripe Grape berries Lamda Zap II Library | INRA | Fruit | 86 |
| 154028 | CabSau Berry Postveraison Stage 36 (POSu0036) | CSIRO | Fruit | 81 |
| 172035 | An expressed sequence tag database for abiotic stressed berries of Vitis vinifera var. Chardonnay | University of Nevada | Berry | 21384 |
| 172296 | Cabernet Sauvignon Leaf-CA48EN | UC Davis | Berry | 2051 |
| 172297 | Cabernet Sauvignon Berry - CAB2SG | UC Davis | Berry | 4429 |
| 172530 | Cabernet Sauvignon Berry Stage I - CAB3 | UC Davis | Berry | 3414 |
| 172531 | Cabernet Sauvignon Berry - CAB4 | UC Davis | Berry | 3836 |
| 173682 | CabSau Berry Fruit Set Stage 28 (PREu0028) | CSIRO | Berry | 36 |
| 174835 | mRNA from grapevine berries collected 12 weeks post flowering | University Stellenbosch | Berry | 7 |
| 175790 | Vitis vinifera Cabernet-Sauvignon fruit | INRA | Fruit | 35 |
| 167554 | cDNA SSH library of leaves from two-month-old Vitis vinifera seedlings | University of Neuchatel | Leaf | 193 |
| 170112 | An expressed sequence tag database for abiotic stressed leaves of Vitis vinifera var. Chardonnay | University of Nevada | Leaf | 24400 |
| 171899 | Cabernet Sauvignon Leaf - CA12LI | UC Davis | Leaf | 1924 |
| 171900 | Cabernet Sauvignon Leaf - CA22LI | UC Davis | Leaf | 1541 |
| 171901 | Cabernet Sauvignon Leaf - CA12EI | UC Davis | Leaf | 4121 |
| 172294 | Cabernet Sauvignon Leaf - CA23EI | UC Davis | Leaf | 2004 |
| 172295 | Cabernet Sauvignon Leaf - CA32EN | UC Davis | Leaf | 2669 |
| 172476 | Cabernet Sauvignon Leaf - CA48LN | UC Davis | Leaf | 2248 |
| 172477 | Cabernet Sauvignon Leaf - CA41LN | UC Davis | Leaf | 1146 |
| 175791 | Vitis vinifera Cabernet-Sauvignon leaf | INRA | Leaf | 27 |
| 737766 | Vitis vinifera cv. Pinot Noir PN162 leaves cDNA library | Centre National de Sequencage, Genoscope | Leaf | 30172 |
| 739408 | Vitis vinifera cv. Pinot Noir PN40024 leaves cDNA library | Centre National de Sequencage, Genoscope | Leaf | 9645 |
| 739410 | Vitis vinifera cv. Pinot Noir PN40024 leaves+petioles cDNA library | Centre National de Sequencage, Genoscope | Leaf/Petioles | 20 |
| 150596 | Cab Sauv pericarp non-normalized (WIN01) | University of British Columbia | Pericarp | 3980 |
| 150599 | Cab Sauv pericarp normalized (WIN04) | University of British Columbia | Pericarp | 5342 |
| 150601 | Cab Sauv seed normalized (WIN06) | University of British Columbia | seed | 1379 |
| 150604 | Cab Sauv pericarp normalized (WIN09) | University of British Columbia | Pericarp | 1392 |
| 150605 | Muscat Hamburg post-veraison pericarp normalized (WIN10) | Centro Nacional de Biotecnologia, CSIC | Pericarp | 8873 |
| 150598 | Cab Sauv seed normalized (WIN03) | University of British Columbia | Seed | 1222 |
| 150601 | Cab Sauv seed normalized (WIN06) | University of British Columbia | Seed | 1379 |
| 150603 | Cab Sauv seed normalized (WIN08) | University of British Columbia | Seed | 9287 |
| 154340 | Mixture of buds, little clusters and fruits (C1) | Universidad Tecnica Federico Santa Maria | Fruit - bud - clusters | 1688 |
| 169138 | Grape cv. Xiahei flower and developing fruit | Nanjing Agricultural University | Flower and Fruit | 10 |
| 173620 | Vitis vinifera cv. cabernet sauvignon Stem - CAST | UC Davis | Stem | 4700 |
