# Supplementary material for: De Novo Sequencing, Assembly, and Analysis of the Root Transcriptome of Persea americana (Mill.) in Response to Phytophthora cinnamomi and Flooding
Source: PLoS One. 2014 Feb 10;9(2):e86399. doi: 10.1371/journal.pone.0086399 (PMC3919710; doi:10.1371/journal.pone.0086399)
Supplement: Table S4 — Putative stress-responsive genes present within the Persea americana dataset. (DOCX) [file pone.0086399.s007.docx]

Table S4. Putative stress-responsive genes present within the *Persea americana* dataset.

| **Contig** | **Gene name** | **EC number** |
| --- | --- | --- |
| 00734, 00748, 00757,01782, 02182, 03305, 00082, 00188, 00336 | AP2/ERF domain-containing transcription factor | EC:3.2.1.0; EC:2.4.1.207 |
| 00786 | ap2-like ethylene-responsive transcription factor at2g41710-like |  |
| 02493, 03143 | ethylene-responsive transcription factor RAP2-7-like |  |
| 03305 | AP2-related transcription factor/ ethylene responsive protein |  |
| 04494 | ethylene responsive transcription factor 3a |  |
| 01040 | WRKY1 |  |
| 03362, 04304 | WRKY2-like |  |
| 04218 | WRKY3-like |  |
| 01318 | Dof-type zinc-finger protein |  |
| 1303 | PHD finger protein |  |
| 00206, 00816, 06878 | bzip transcription factor |  |
| 02977 | bzip 53 transcription factor |  |
| 03280 | bzip transcription factor bzip52 |  |
| 03418, 04919, 05237 | bzip transcription factor bzip114 |  |
| 05116 | bzip transcription factor bzip80 |  |
| 05860 | bzip transcription factor-like protein |  |
| 03280, 04651 | TGA transcription factor |  |
| 00547 | myb transcription factor myb93 |  |
| 00608, 03369 | r2r3-myb transcription factor |  |
| 02717 | myb transcription factor myb178 |  |
| 03985, 04626, 05714 | myb transcription factor |  |
| 05121, 06590 | mybr domain class transcription factor |  |
| 03450, 01168, 06475 | nac domain protein |  |
| 05487 | nac domain containing protein 45 |  |
| 06533, 00313, 00314, 00861, 00956, 01964, 02265, 01167 | nac domain ipr003441 |  |
| 00414 | nac protein 1 |  |
| 01009 | nac domain containing protein 87 |  |
| 02411 | nac transcription factor-like 9 |  |
| 00832 | vesicle transport v-snare 13 |  |
| 01747, 02777 | snare protein |  |
| 02967 | golgi snare 12 protein |  |
| 04832 | bet1-like snare 1-1 |  |
| 02858, 03491, 04337 | linoleate 13s-lipoxygenase | EC:1.13.11.12 |
| 00670, 03745 | allene oxide synthase | EC:4.2.1.92 |
| 06180, 01851 | allene oxide cyclase | EC:5.3.99.6 |
| 00307, 00651, 02176, 04379 | 12-oxophylodienoate reductase | EC:1.3.1.42 |
| 02547, 00123 | 3-ketoacyl- thiolase | EC:2.3.1.16 |
| 00222, 01151, 02379 | f-box family protein |  |
| 00576 | f-box protein at1g67340-like |  |
| 01831 | f-box protein pp2-a13 |  |
| 00402, 00207, 00233 | s-adenosylmethionine synthase 5 | EC:2.5.1.6 |
| 06266, 04547, 04234 | ankyrin repeat family protein |  |
| 04761 | ankyrin repeat-containing |  |
| 04014 | histidine kinase 3 | EC:3.1.3.16 |
| 05612, 07123 | histidine kinase cytokinin receptor |  |
| 00644, 03661, 04177, 04368, 05227 | prolyl 4-hydroxylase alpha | EC:1.14.11.2,EC:1.14.11.0 |
| 00998 | calcineurin b-like protein-interacting protein kinase | EC:2.7.11.0 |
| 04353 | phospholipase c | EC:3.1.4.11 |
| 04114, 03975 | nadph oxidase | EC:1.6.3.1;EC:1.11.1.7 |
| 04277 | rho gtpase |  |
| 00804, 02081, 06163, 00585 | phosphoenolpyruvate carboxylase | EC:1.3.1.74;EC:4.1.1.31 |
| 00469, 00762, 01869, 05606, 07046 | malate dehydrogenase | EC:1.1.1.37 |
| 00298, 02325 | nitrate reductase | EC:1.7.1.1; EC:1.7.1.2 |
