# Supplementary material for: De Novo Sequencing, Assembly, and Analysis of the Root Transcriptome of Persea americana (Mill.) in Response to Phytophthora cinnamomi and Flooding
Source: PLoS One. 2014 Feb 10;9(2):e86399. doi: 10.1371/journal.pone.0086399 (PMC3919710; doi:10.1371/journal.pone.0086399)
Supplement: Table S5 — Primer sequences for genes analysed in expression analysis. (DOCX) [file pone.0086399.s008.docx]

**Table S5.** Primer sequences for genes analysed in expression analysis.

| **Gene** | **Forward primer (5’-3’)** | **Reverse primer (5’-3’)** | **Product size (bp)** |
| --- | --- | --- | --- |
| Actin | CCAAGCAGCATGAAGATAAAGGT | CACATCTGTTGGAAGGTGCTC | 103 |
| 18S | GTTACTTTAGGACTCCGCC | TTCCTTTAAGTTTCAGCCTTG | 90 |
| Alpha-1 tubulin | AAGGATTATGAGGAGGTTG | ATCAGCCACATTCTCTTC | 87 |
| SucS | CATACATCAAACCGTGAGATCCA | GTACTACTTGCAACCAGCGT | 120 |
| NSH | TCAAGATGACCTGTGAAGCA | CCTTCTTAAGATGAACTGAACCC | 101 |
| PDC | GAGGGTGCAAACACAATGGA | CGCAATACAGTAACCCAAACCA | 116 |
| Endo | ATCACCAACATCATCAAC | CTCTTGTAGAAGCCAATG | 83 |
